# The Association of *PTPN22* R620W Polymorphism Is Stronger with Late-Onset AChR-Myasthenia Gravis in Turkey

**DOI:** 10.1371/journal.pone.0104760

**Published:** 2014-08-13

**Authors:** Gizem A. Kaya, Ayse N. Coşkun, Vuslat Yılmaz, Piraye Oflazer, Yeşim Gülsen-Parman, Fikret Aysal, Rian Disci, Haner Direskeneli, Alexander Marx, Feza Deymeer, Güher Saruhan-Direskeneli

**Affiliations:** 1 Istanbul University, Istanbul Medical Faculty, Istanbul, Turkey; 2 Istanbul University, Istanbul Medical Faculty, Department of Physiology, Istanbul, Turkey; 3 Istanbul University, Istanbul Medical Faculty, Department of Neurology, Istanbul, Turkey; 4 Bakirkoy Hospital for Psychiatric and Neurological Diseases, Istanbul, Turkey; 5 Istanbul University, Istanbul Medical Faculty, Department of Biostatistics, Istanbul, Turkey; 6 Marmara University, Faculty of Medicine, Division of Rheumatology, Istanbul, Turkey; 7 University of Heidelberg, University Medical Centre Mannheim, Institute of Pathology, Mannheim, Germany; University of Sydney, Australia

## Abstract

A functional single nucleotide polymorphism (SNP) of the *PTPN22* gene encoding a protein tyrosine phosphatase has been associated with autoimmune disorders including myasthenia gravis (MG). As the *PTPN22* R620W polymorphism has a wide variation of allele frequencies among different populations, this polymorphism was investigated in MG in Turkey. An emphasis is put on MG subgroups according to autoantibody (Abs) production and presence of thymoma. DNA samples from 416 patients with clinically diagnosed generalized MG (231 with Abs to acetylcholine receptor, AChR-MG), 53 with Abs to muscle-specific kinase (MuSK-MG), 55 patients with no detectable Abs (SN-MG), 77 patients with thymoma (TAMG) and 293 healthy controls (HC) were genotyped for the SNP (*PTPN22* R620W, C1858T, rs2476601). The *PTPN22* T allele was increased in AChR-MG patients (odds ratio [OR]: 2.5, 95%CI: 1.2–5.1). The association was stronger in late disease-onset AChR (LOMG, OR: 3.1, 95%CI: 1.2–8.2). MuSK-MG, SN-MG and TAMG groups did not carry the variant allele more frequently than the HC. In contrast to findings in other autoimmune diseases, the distribution of the *PTPN22* polymorphism in this population provides a susceptibility marker for AChR-MG. The strongest association is detected in patients with LOMG.

## Introduction

Acquired myasthenia gravis (MG) is a rare autoimmune disease which is clinically characterized by fatigability and weakness of striated muscles. The symptoms of MG are mediated mainly by pathogenic auto-antibodies (Abs) directed against the nicotinic acetylcholine receptor (AChR). These disease specific anti-AChR Abs are detected in the majority (80–85%) of the patients [1], [2]. In a subgroup of MG patients without conventional anti-AChR Abs, Abs against the muscle-specific kinase (MuSK) are detected [3]. Recently, the agrin receptor low-density lipoprotein receptor-related protein 4 (LRP4) has been identified as a novel target in a 2–50% of AChR and MuSK double seronegative patients [4], [5], [6]. Subsequently anti-agrin antibodies has also been reported in a small proportion of AChR-MG and triple seronegative patients [7]. Furthermore, anti-AChR antibody positive MG (AChR-MG) appears as a heterogeneous disease subset with or without thymoma and differences related to age of disease onset. The cut-off age between late-onset (LOMG) and early-onset MG (EOMG) has been shifted from 40 years [8] to 50 [9], [10], [11] and even to 60 years on the basis of clinical, histological and immune-genetic data [12], [13], [14].

The initiation of the auto-immune response is not understood in MG. Dependence of antibody producing B cells on T cells as well as thymic changes indicate a pivotal involvement of T cells in the disease pathogenesis. Altered T cell receptor (TCR) signaling has been recognized as a risk factor for other autoimmune diseases. Altering TCR signaling may predispose to diseases by changing thymic selection, T helper or T regulatory (Treg) cell activity [15]. Protein tyrosine phosphatase non receptor 22 gene (*PTPN22*) encodes a lymphoid-specific phosphatase that is involved in terminating TCR signaling and calibrating the T cell activation threshold. A single nucleotide polymorphism (SNP) of *PTPN22* causing an amino acid change (R620W, C1858T, dbSNP reference: rs2476601) has been shown to affect the interaction of this protein phosphatase with Src family kinases in T cell activation [16]. Individuals carrying the variant allele of *PTPN22* (T allele encoding W620) may have changes in the threshold for thymic selection and be prone to autoimmunity. However, the mechanism of action remains to be clarified and both gain and loss of function data have been reported [17], [18], [19].

MG was shown to be associated with *PTPN22* R620W polymorphism similar to several other autoimmune diseases. The polymorphic allele was increased in the non-thymoma MG patients without anti-titin antibodies (ATA) (odds ratio [OR]: 1.97) [20]. In subsequent studies on Swedish, German and Hungarian MG patients, this *PTPN22* variant was associated with AChR-MG [21], [22], [23] and with thymoma-associated MG (TAMG) in one study [21]. Two meta-analyses of published MG data demonstrated a combined OR of 1.53 and 1.64 for this SNP [24], [25]. The first genome-wide association study published in MG recently revealed the expected association with *PTPN22* (rs2476601; OR: 1.71) in a larger sample of EOMG cases from European populations [15]. This association could not be replicated in an Italian population that has been shown to be genetically similar to the population of Turkey and other Mediterranean countries [26]. Because the P*TPN22* R620W polymorphism demonstrates a wide variation among different populations, with the highest polymorphic allele presence being in Scandinavia (15%) yet absent in Asian and African populations [15], [27], this polymorphism is being investigated in this study as a susceptibility marker in MG patients and within heterogeneous disease subgroups from Turkey.

## Materials and Methods

### Patients and Controls

Four hundred sixteen MG patients (265 women, 64%) were included in the study group. All patients were diagnosed as having generalized MG based on clinical criteria. Among the patients with MG in this study, 19% had thymoma (TAMG). To investigate potential associations of disease subgroups, patients were separated on the basis of antibody profile and by age of onset. Regarding the age which separates EOMG from LOMG, 50 years of age was applied (LOMG, ≥50 years) [9].

A group of 293 volunteers were investigated as healthy controls (HC) (146 women, 50%). All individuals donated blood after being informed about the study and written informed consent was obtained for genetic analysis. The Ethical Review Board of Istanbul Medical Faculty approved the study. The main characteristics of the patients and HC are summarized in Table 1.

**Table 1:** Gender distribution in patients with myasthenia gravis (MG), anti-AChR antibody positive MG (AChR-MG) with early disease onset (EOMG<50 years of age) and late disease onset (LOMG≥50 years of age), anti-MuSK antibody positive MG (MuSK-MG), MG without any of these two antibodies (SN-MG), thymoma associated MG (TAMG) and healthy controls (HC).

	♀	%	♂	%	Total (N)
**MG**	265	63,7	151	36,3	416
**AChR-MG**	148	64,1	83	35,9	231
** EOMG**	126	73,7	45	26,3	171
** LOMG**	22	36,7	38	63,3	60
**MuSK-MG**	39	73,6	14	26,4	53
**SN-MG**	35	63,6	20	36,4	55
**TAMG**	43	55,8	34	44,2	77
**HC**	146	49,8	147	50,2	293

### Antibody Determinations

Anti-AChR and anti-MuSK Abs were detected by radioimmunoprecipitation assay (RIA) using commercial kits (All from DLD Diagnostika GmBH, Hamburg, Germany).

### *PTPN22* Genotyping

DNA samples from patients and HC were genotyped by polymerase chain reaction (PCR) based on restriction fragment length polymorphism (RFLP) analysis for the SNP (rs2476601, R620W, C1858T, C→T) of *PTPN22* gene. DNA was amplified using the forward primer 5′GGC CTC AAT GAA CTC CTC AA 3′ and reverse primer 5′AAT GTT GCT TCA ACG GAA TTT 3′ [28]. PCR amplification was carried out in (NH_4_) buffer with 2 mM MgCl_2_, 200 µM dNTP, 0.4 µM of each primer, 100 ng of genomic DNA and 0.75 IU of *Taq* polymerase (Fermentas, Germany). The cycling parameters were as follows: initial denaturation step of 2 min at 95°C, 30 cycles of 30 sec at 95°C, 45 sec at 59°C, 45 sec at 72°C and final extension step of 2 min at 72°C. The polymorphism was identified by *Xcm* I (New England Biolabs Inc., UK) digestion for 4 hours at 37°C and visualized on 3% agarose gel stained with ethidium bromide. Repeated typing was performed on several samples with identical results.

### Statistical Analysis

The distribution of the polymorphism in the patient and HC groups was determined by counting. Genotype distributions were compared between groups and subgroups by chi-square and Fisher's exact tests where appropriate. The strength of associations was estimated by odds ratio (OR) with 95% confidence intervals (95% CI). P values less than 0.05 were considered significant. Logistic regression analysis was performed for the interaction of sex and disease onset (EOMG and LOMG) in the AChR-MG group.

## Results

The distribution of the *PTPN22* C1858T polymorphism (C→T) revealed that the genotype frequencies were in Hardy-Weinberg equilibrium in the HC. In this group, the frequency of heterozygous genotype (TC) was detected only in 4.1% (12/293) of donors corresponding to a minor allele frequency (MAF) of 0.02. Homozygous TT genotype was not found in the HC group (Table 2).

**Table 2:** The distribution of the *PTPN22* (rs2476601 T→C) genotype frequencies in healthy controls (HC) and myasthenia gravis (MG) patients.

rs 2476601 (T→C)	N	CC	%	TT+CT	%	p	OR (95%CI)
**MG**	416	385	92.5	31	7.5	0.08	1.9 (1.0–3.7)
**AChR-MG**	231	209	90.5	22	9.5	0.013	2.5 (1.2–5.1)
** EOMG**	171	156	91.2	15	8.8	0.04	2.3 (1.0–4.9)
** LOMG**	60	53	88.3	7	11.7	0.03	3.1 (1.2–8.2)
**MuSK-MG**	53	51	96.2	2	3.8	NS	
**SN-MG**	55	51	92.7	4	7.3	NS	
**TAMG**	77	74	96.1	3	3.9	NS	
**HC**	293	281	95.9	12	4.1		

AChR-MG, MuSK-MG, SN-MG and thymoma associated MG (TAMG) subgroups are compared with HC separately and only the significant p value is listed. OR: Odds ratio, 95% Confidence interval (CI), (NS: not significant compared to HC).

The *PTPN22* C1858T genotypes implicated significant differences between groups (Table 2). The polymorphic T allele encoding tryptophan (W) at the 620.residue of PTPN22 was more prevalent in the whole MG group without reaching statistical significance level. To study a possible effect of this polymorphism in different antibody producing disease subtypes, patients from relatively small subgroups such as MuSK-MG, SN-MG and TAMG were particularly recruited in addition to the main group, namely AChR-MG.

AChR-MG patients, excluding TAMG patients, carried the *PTPN22* T allele significantly more frequently than HC (9.5 vs. 4.1%, OR: 2.5, 95% confidence interval [CI]: 1.2–5.1, p = 0.013, Table 2). The distribution of *PTPN22* alleles was not significantly different between TAMG and HC groups. Only 3 out of the 77 TAMG patients carried the polymorphic *PTPN22* T allele as one copy only (Table 2).

Similarly, only 3.9% of MuSK-MG patient group carried the polymorphic allele, based on a proportion of 13% of the whole MG group. Both anti-MuSK and anti-AChR Abs negative, SN-MG patients made up 13% of the study group and carried the *PTPN22* T allele in 7.3% without a significant difference to HC (Table 2).

When the AChR-MG patients were subgrouped according to 50 years of age as the cut-off for disease onset, the *PTPN22* T allele was more strongly associated with LOMG. The T allele was significantly more frequent in the smaller LOMG group (OR: 3.1, 95%CI: 1.2–8.2, p = 0.03), but the association was also evident in EOMG (OR: 2.3, 95%CI: 1.0–4.9, p = 0.04, Table 2). The difference between LOMG and EOMG was not significant. Confirming the previous observation of imbalanced sex distribution in disease-onset subgroups [14], [29], the proportions of women and men were significantly different in the EOMG and LOMG groups (p<0.001). In LOMG, men prevailed (63%) whereas women dominated the EOMG group (74%). To delineate the interaction between disease onset and sex, logistic regression analysis was performed (Table 3). In logistic regression analysis, LOMG (p = 0.02, OR: 3.4) was significantly associated with the presence of T allele irrespective of the sex. The associations of *PTPN22* polymorphism with EOMG (p = 0.10, OR: 1.95) and being women (p = 0.10, OR: 1.95) in AChR-MG patients were not statistically significant with this analysis.

**Table 3:** The logistic regression analysis of the *PTPN22* (rs2476601 T→C) genotype frequencies with sex (women and men) and disease onset (early onset: EOMG and late onset: LOMG) in anti-AChR antibody positive MG (AChR-MG) with reference to healthy controls (HC).

		B	p	OR	95% C.I.	
					Lower	Upper
**Sex**	Women	0,67	0,10	1,95	0,88	4,34
	Men			1,0		
**Group**	EOMG	0,67	0,10	1,95	0,88	4,34
	LOMG	1,23	0,02	3,41	1,27	9,18
	HC			1,0		

OR: Odds ratio, 95% Confidence interval (CI).

## Discussion

The *PTPN22* R620W polymorphism is accepted as a general risk factor for autoimmune diseases with prominent production of auto-antibodies [15]. MG, being a prototypic auto-antibody mediated disease, has also been one of the diseases showing association with the *PTPN22* R620W polymorphism. A relatively strong association of the polymorphic *PTPN22* 1858 T allele was demonstrated with AChR-MG also in this population from Turkey, where the MAF of the polymorphic allele was as low as 2%. By stratification of the patients, we also showed that the association was stronger particularly in LOMG than in the whole AChR-MG cohort.

Among the reported *PTPN22* associations with MG, several subgroup analyses have been described in order to cover and delineate the diverse clinical or immunological subgroups of MG. Categorizing the disease according to thymoma and ATA, Vandiedonck et al. found non-thymoma patients without ATA (predominantly positive for anti-AChR Abs) as the susceptible group for this association with an OR of 1.97 [20], which was later replicated in a relatively small cohort of German and Hungarian populations (OR: 2.1) [22]. Similarly, Swedish patients with anti-AChR antibodies and accompanying thymic hyperplasia were most susceptible for this association (OR: 1.96) [23]. In another study from Germany, EOMG but also TAMG subgroups had increased frequencies of the *PTPN22* polymorphic allele compared with ethnically matched controls (OR: 2.4 and 2.5) [21]. Based on the recently performed meta-analyses and genome-wide results, the most robust conclusion is that the minor allele (T) of *PTPN22* polymorphism is associated with the presence of anti-AChR Abs and with the absence of thymoma in MG [15], [24], [25]. The main exception to this association was data derived from an Italian population where no association was found with MG. In contrast, the findings of this study demonstrated a relatively strong association of *PTPN22* with AChR-MG providing an OR similar to other Caucasian populations and even higher than the cumulative value of the meta-analyses. This discrepancy between Italian and Turkish populations in susceptibility to MG is unexpected. The Italian population is reported to be genetically different from the mentioned European populations, but similar to populations from other Mediterranean countries including Turkey [24]. Previous studies on other diseases including our study on MuSK-MG have emphasized the similar genetic backgrounds of Mediterranean populations [30], [31], [32], [33]. The heterogeneity in the samples selected for the studies from Italy and from Turkey may account for this difference [24].

Another finding of this study was the stronger association of this polymorphism with LOMG than the EOMG group which was not reported in other populations. As the previous studies have focused mainly on EOMG, the extension of association to LOMG may provide a novel aspect for this disease subgroup. However, we have also observed an inbalance of the sex distribution in this relatively smaller subgroup of AChR-MG and the association with the *PTPN22* R620W polymorphism was even stronger in women of LOMG group compared with healthy women. Thus, the previous observation of stronger association of HLA-B8 with women in MG [15], [34] extends to another locus, *PTPN22*. This difference of *PTPN22* in women and men was not observed previously in the genome-wide association study [15], but similar findings have been proposed previously in rheumatoid arthritis (RA) for *PTPN22* [28], [35]. In the present study, however, the effect of sex did not persist after the regression analysis. Further studies with higher sample numbers are required to verify this finding.

Data from this study did not provide evidence for an increase of the *PTPN22* T allele in TAMG, similar to the French and Swedish cohorts [20], [23] and at variance with a German cohort [21]. Several studies have shown that LOMG and TAMG have similarities in autoantibody spectrum [14]. The polymorphic *PTPN22* allele frequency in the rare subgroup of MuSK-MG was as low as in the HC and this finding confirms the single previous result in MuSK-MG reported from Italy [24]. The lack of association with both TAMG and MuSK-MG emphasizes the differences between genetic backgrounds of these subgroups and AChR-MG.

The variable distribution of autoimmunity-associated genes might hint to the different frequencies of diseases in different ethnical populations. Among the non-HLA genetic associations with autoimmune diseases, the *PTPN22* R620W gene polymorphism was shown to be a major risk factor in association studies of several diseases in Caucasian populations including North American, Spanish, British, Dutch, French-Canadian and Swedish patients [36]. However, there were considerable ethnical differences in the polymorphic allele frequencies of *PTPN22* R620W polymorphism in different populations with a North-South gradient ranging between 15.5% and 2.1% in Europe [15], [27]. The frequency of the *PTPN22* polymorphism was also relatively low in the healthy population from Turkey in the present data as well as in another sample from Turkey [37]. Our previous results on the absence of the association with this *PTPN22* polymorphism in other autoimmune diseases such as RA and Takayasu's arthritis [38], [39] in this population underlines the specificity of the current finding in AChR-MG, also supporting the relationship with specific humoral autoimmunity.

Several association studies have shown genetic heterogeneity of MG as in HLA associations. The current study provides a confirmation for an association with AChR-MG in a relatively small sample size in a population with a low frequency of this polymorphism. Moreover, the findings identify *PTPN22* R620W polymorphism as the strongest susceptibility marker for LOMG among all other MG subgroups and provide a hint for a sex related difference in susceptibility for AChR-MG. Although no functional implications can be concluded from the data, further analysis of the *PTPN22* polymorphism in larger samples and at the cellular level is warranted in disease groups.
